# Physiologic Solutions are Superior to Normal Saline in Critically Ill Patients: PRO

**DOI:** 10.34067/KID.0000000629

**Published:** 2025-03-20

**Authors:** Luis A. Juncos, Michael J. Connor

**Affiliations:** 1 Critical Care, Fresenius Medical Care, Waltham, Massachusetts; 2 Department of Medicine, University of Arkansas for Medical Sciences, Little Rock, Arkansas; 3 Department of Medicine, Emory University School of Medicine, Atlanta, Georgia

**Keywords:** AKI

Fluid therapy is essential in treating critically ill patients with conditions such as shock, sepsis, organ failure, and volume depletion.^1^ While 0.9% normal saline (NS) has long been the standard because of its widespread availability and simplicity, evidence now questions its appropriateness for such patients. Concerns include its link to hyperchloremic acidosis and kidney dysfunction, leading to a shift toward physiologic or balanced crystalloids like lactated Ringer's and Plasma-Lyte.^2^ These fluids, designed to mimic plasma composition, offer potential physiologic benefits, although the impact on clinical outcomes remains debated. This article argues that balanced crystalloids are superior to NS because of their physiological properties, lower risks of hyperchloremia and acidosis, and improved outcomes in critical care.

## Physiological Considerations

NS is an unbuffered solution with 154 mEq/L of sodium and chloride, with chloride levels exceeding the normal plasma range of 94–111 mEq/L. This supraphysiologic chloride content can cause adverse effects. For instance, Wilcox's lab showed NS induces afferent arteriolar vasoconstriction, reducing kidney perfusion and GFR, which may heighten the risk of AKI, especially in critically ill patients, who often have compromised kidney function. NS also promotes non-anion gap hyperchloremic metabolic acidosis, leading to renal and cardiovascular dysfunction, electrolyte imbalances, and impaired oxygen delivery.^3^ In addition, NS may impair immune and neurological functions, increasing the risk of infections and altered mental status. These risks do not occur uniformly across all patients, rather they depend on the volume of fluid administered—typically requiring multiple liters for adverse effects to manifest—and the patient's underlying susceptibility, with critically ill patients requiring larger volumes being the most vulnerable.

**Table 1:** Composition and characteristics of commonly used intravenous solutions

Solution	Na^+^ (mEq/L)	K^+^ (mEq/L)	Cl^−^ (mEq/L)	HCO_3_− or Lactate (mEq/L)	Ca^++^ (mEq/L)	Mg^++^ (mEq/L)	Glucose (g/L)	Osmolarity (mOsm/L)
Human plasma	136–145	3.5–5	94–105	24 (HCO_3_−)	2–2.5	1.5–2	0.9 (fasting)	280–295
0.9% NaCl	154	0	154	0	0	0	0	308
Lactated Ringer's	130	4	109	28 Lactate	3	0	0	273
Plasma-lyte A	140	5	98	27 Acetate+gluconate	0	3	0	294
Normosol-R	140	5	98	27 Acetate+gluconate	0	3	0	294
D5W	0	0	0	0	0	0	50	252
0.45% NaCl	77	0	77	0	0	0	0	154
D5 1/2 NS	77	0	77	0	0	0	50	406

NS, normal saline.

By contrast, balanced crystalloids have more physiologically appropriate electrolyte compositions (Table 1). Their lower chloride concentration and the inclusion of a base (*e.g*., acetate, lactate, or gluconate) help maintain acid-base balance and reduce the risk of metabolic acidosis. The presence of other electrolytes supports better overall balance, especially in patients at risk for hypokalemia or hypocalcemia. By avoiding hyperchloremia and its complications, balanced crystalloids offer superior support, particularly in critically ill patients with renal dysfunction, sepsis, or shock.

## Clinical Evidence Supporting Physiologic Solutions

Evidence supporting the superiority of balanced crystalloids in critically ill patients has been strengthened by several randomized controlled trials (RCTs) and large observational studies. The SALT-ED trial,^4^ a single-center multiple cross-over study of over 13,000 adults treated in the emergency department and hospitalized, compared balanced crystalloids with NS. While there was no significant difference in hospital-free days at 28 days (primary outcome), balanced crystalloids were associated with a lower incidence of major adverse kidney events, including death, new KRT, or persistent renal dysfunction, compared with NS (4.7% versus 5.6%).

The SMART trial,^5^ a pragmatic, cluster-randomized study of 15,802 critically ill patients, found lower composite major adverse kidney event rates at 30 days in the balanced solution group. The incidence of death, new KRT, or persistent renal dysfunction was 14.3% in the saline group versus 13.8% in the balanced solution group. While these differences are small, the results are clinically meaningful because of the large sample size and inclusion of high-risk patients where balanced crystalloids may be advantageous.

The benefits of balanced crystalloids may extend beyond the ICU. The BEST-Fluids trial,^6^ a multi-center RCT, showed that balanced crystalloids reduced delayed graft function in kidney transplant recipients compared with NS (30% versus 40%), leading to the recommendation that they become the standard for fluid management in deceased donor kidney transplants. In addition, Shaw *et al.* found that patients receiving only balanced crystalloids during major abdominal surgery had fewer postoperative complications and reduced hospital resource use (*e.g*., fewer blood gas checks, transfusions, KRT) than those who received NS.^3^

Not all studies have supported the superiority of balanced crystalloids. The SPLIT trial,^7^ a prospective, blinded, cluster-randomized double cross-over study, found no difference in AKI rates between groups, but this was a low-risk population that received small fluid volumes. The BaSICS trial^8^ also found no effect of fluid type or administration rate on mortality or AKI rates. However, the high proportion of elective surgical patients in the trial lowered the overall mortality (the observed mortality was lower than expected), reducing the study's power to detect differences. The PLUS trial,^9^ a double blind RCT conducted in 53 ICUs across Australasia, also found no difference in 90-day mortality or secondary outcomes (new KRT or changes in creatinine levels) despite receiving more generous fluid volumes (median 3.9 L). However, the frequent use of pre-enrollment saline and significant cross-over may have reduced the difference in chloride exposure between groups, thus masking any protective effect of balanced solutions. Moreover, their results indicated that there could have been an approximately 3% change in the risk of death or new KRT that was undetected. Despite the negative findings of these trials, a meta-analysis that included all these trials concluded there is a high probability that balanced solutions reduce mortality among critically ill adults, with possible subgroup effects.^10^

## Economic Implications

Although balanced crystalloids are more expensive than NS (approximately $1.50–$2.00 more per liter), the potential savings from reduced complications and shorter hospital stays may easily offset the cost difference. Preventing AKI alone can save $5000–$10,000 per hospitalization, not including long-term KRT costs ($70,000–90,000 per year). In addition, avoiding NS-related complications could reduce costs by $2000–$10,000 for procedures like inpatient KRT, blood transfusions, and blood gas draws. Balanced crystalloids may also shorten ICU stays, which cost $2000–$4000 per day. From a health care perspective, these savings far outweigh the initial higher cost.

## Counterarguments

Advocates for NS emphasize its long-standing use, affordability, and universal availability as key advantages. They note its effectiveness in expanding extracellular fluid volume and stabilizing BP in hypovolemic patients. With decades of use, NS is seen as a reliable option with a reasonable safety profile. However, this view overlooks growing evidence of its risks, particularly in critically ill patients at high risk for adverse events and organ failure. The potential for hyperchloremic metabolic acidosis and kidney damage in vulnerable populations, raises concerns. While NS is appropriate in specific cases, such as hyponatremia, hypochloremia, and traumatic brain injury (as suggested by the BaSICS and PLUS studies), balanced crystalloids offer a more physiologically suitable option for most other patients. The shift toward balanced crystalloids reflects their ability to reduce complications and better match the body's natural electrolyte balance.

## Conclusions

The preponderance of evidence supports the use of balanced crystalloids over NS, in critically ill patients requiring large-volume resuscitations. Balanced crystalloids offer a more physiologically appropriate composition, reducing the risks of renal hypoperfusion and hyperchloremic metabolic acidosis. While mortality benefits remain inconclusive, several large studies have shown a reduction in major adverse kidney events, especially in patients prone to AKI. Importantly, there is no evidence suggesting increased harm from balanced solutions, except in cases of acute traumatic brain injury. Despite their slightly higher upfront cost, balanced crystalloids may reduce overall health care expenses by preventing complications, decreasing hospital resource utilization, and shortening hospital stays, making them a compelling choice for high-risk patients. While NS remains valuable in specific situations (*e.g*., traumatic brain injury, hyponatremia, or hypochloremia), the evidence increasingly favors balanced fluids for most other critically ill patients. Thus, routine NS use in critically ill patients should be reconsidered, with balanced solutions emerging as the preferred choice. Continued research is necessary to refine the use of fluids in specific patient populations and further establish the long-term benefits of balanced solutions.
